# Synthesis of P-/N-Containing Bamboo-Activated Carbon toward Enhanced Thermal Stability and Flame Retardancy of Polylactic Acid

**DOI:** 10.3390/ma15196802

**Published:** 2022-09-30

**Authors:** Ningning Yin, Jinhuan Zhong, Huayu Tian, Zenan Zhou, Weijun Ying, Jinfeng Dai, Wenzhu Li, Wenbiao Zhang

**Affiliations:** 1 College of Chemistry and Materials Engineering, Zhejiang Agriculture and Forestry University, Hangzhou 311300, China; 2 Jiyang College, Zhejiang Agriculture and Forestry University, Shaoxing 311800, China

**Keywords:** bamboo-activated carbon, grafting, thermal stability, flame retardancy, polylactic acid

## Abstract

A P-/N-containing bamboo-activated carbon (BACm) was successfully synthesized by steam activation of bamboo charcoal and chemical grafting to as-prepared activated carbon using the reaction of phosphoric acid and urea. Characterizations of BACm presented a synergistic grafting of P and N elements to the BAC surface. The BACm was further loaded in a polylactic acid (PLA) matrix to prepare BACm/PLA composites. Mechanical strength study showed tensile strength dropped from 75.19 MPa to 61.30 MPa, and tensile modulus from 602.49 MPa to 375.56 MPa, suggesting a rigidity reduction and deformation resistance enhancement owing to the roughened surface of BACm that interlocked with the polymer. The thermogravimetric analysis showed that the carbon residue rate of BACm dramatically fell to 49.25 wt.% in contrast to 88.28% for the control BAC, and cone calorimeter measurements confirmed the enhancement of flame retardancy of the composites with BACm loading, and the carbon residue rate increased progressively with BACm loading in the composites, notably up to 8.60 wt.% for the BAC/PLA9 composite, which outweighed the theoretical residue rate by more than 50%. The elemental analysis also confirmed rich P/N levels of the dense carbon residue layer that could perform synergistically and effectively in fire suppression. The BACm tended to stimulate the earlier decomposition of the composites and formed a continuous residual carbon layer which functioned as an effective barrier hindering the mass and heat transfer between the combustion zone and the underlying matrix. Moreover, 9 wt.% of BACm loading could attain a V-0 rating (UL94) for the composite with an improved limiting oxygen index up to 31.7%. The biomass-based modified activated carbon in this work could be considered as an alternative flame retardant in polymer applications.

## 1. Introduction

The extensive use of traditional fossil-based polymers has become a major pollutant on land and at sea, which has a great impact on the survival and reproduction of organisms [1,2,3]. The emergence of degradable plastics derived from renewable resources has provided a route to reduce the potential damage to the environment and health, and has gradually been an alternative to conventional polymers in various end uses. Polylactic acid (PLA) is one candidate that has attracted extensive academic interest and industrial attention for its excellent bio-compatibility and biodegradability, favorable processibility and abundance in raw material supply, which makes it ubiquitously used in food packaging, medical products, vehicle parts, 3D printing and the aerospace industry [4,5,6,7]. However, the inadequate thermal stability and easy degradation at high temperatures greatly hamper its perspective applications in electronics and various commercial fields.

Vast research has been conducted to improve and release the issue. Conventional flame retardants, such as phosphate, aluminum hydroxide, talc, etc., are used to enhance the flame retardancy of PLA [8,9,10], and the loading quantity into the polymer formulation is generally quite high, which could jeopardize the composites’ strength and undoubtedly impact its applications, which is not an optimal strategy to improve the thermal performance of PLA at the expense of the mechanical property.

Intumescent flame retardant is considered as an effective additive featured by low smoke and toxicity, and is widely utilized in PLA composites. The traditional intumescent flame retardant includes three parts: carbon, gas and acid sources [11,12]. The system can form a dense carbon layer that acts as a physical barrier, alleviating the heat and mass transfer between condensed phase gases, and therefore protecting the underlying material from heat flow and flame attack. A typical combination of an expansion system is ammonium polyphosphate (APP) and pentaerythritol (PER), in which APP plays the role of acid and gas sources (ammonia precipitation during APP degradation) and PER as a carbon source [13]. Multiple bio-materials such as lignin [14,15] and starch [13,16] have been grafted to the intumescent flame retardant system to improve flame retardancy. Additionally, carbon-based materials, such as graphite [17,18,19,20], graphene [21,22,23] and carbon nanotube [24,25,26] were usually considered outstanding options due to their unique structure (dense networking); nonetheless, the inferior dispersion in matrix and cost concerns significantly dampen the incentive for their commercial development.

Cost-efficient bamboo charcoal, obtained from bamboo and processing residues carbonized in the absence of oxygen, has attracted great research interest. Li et al. [27] contend that mechanical, electrical and thermal properties can be improved with highly filled bamboo charcoal/ultra-high molecular weight polyethylene composites. Qian et al. [28] reported that the impact strength and thermal property of the PLA composite can be reinforced with ultra-fine bamboo charcoal. Sheng et al. [29] fabricated high-toughness PLA/Bamboo cellulose nanowhiskers, and bionanocomposite was obtained by strengthening with silylated ultra-fine bamboo charcoal. Kumar et al. [30] prepared a flexible nanocomposite and a drastic improvement can be seen in the thermal stability and electrical conductivity by adding small amount (<2.5 wt.%) of nano bamboo charcoal reinforcement in the organic PU foam. In the authors’ previous study [31,32], a BC/PLA (25/75) composite had been obtained and the study suggested that the LOI of the composite slightly increased to 23.8 vol.% as compared to 22.0 vol.% of neat PLA, and APP was added to improve the flame retardancy. In addition, chemical modification by phosphate/urea was also adopted to synthesize intumescent bamboo charcoal (BC-m) that claimed an improvement in the flame retardancy of PLA composites, yet it demanded a quite complicated process and maneuverings to obtain an oxidative bamboo charcoal(BC-o) and intumescent bamboo charcoal (BC-m) sample. Moreover, relatively high loading (20 wt.%) of the BC-m into the composites was required to achieve the V-0 level according to te hUL-94 test result, meanwhile, the mechanical property was undermined to a certain extent.

Activated carbon has been widely used in liquid and gaseous purification [33,34], because its abundant pore structure and specific surface area allows itself to adsorb chemicals and provides an excellent support to be modified, and investigations regarding its application in polymeric formulation are also accessible. Gong et al. [35] reported that nickel oxide-loaded activated carbon improved the thermal stability and flame retardancy of polypropylene; Wen et al. [36] found that nanoscale carbon black can promote the degradation of PLA and form a dense carbon layer faster when assisted by nickel oxide. Zhang et al. [37] found that the synergistic effect of activated carbon and molybdenum oxide greatly reduced the total heat release and flue gas release of polyvinyl chloride.

Bamboo charcoal can be steam activated to obtain activated carbon with oxygen-containing functional groups (such as hydroxyl and carboxyl) that can offer reactive sites for adsorption and grafting flame retardants. Green and environmentally friendly phosphoric acid and urea with high P/N content were selected as grafting elements, and bamboo charcoal was designated as a flame retardant carrier to successfully synthesize P-/N-containing samples through adsorption and chemical reaction. It is expected to have excellent flame retardant properties while maintaining non-toxic and biodegradable properties.

## 2. Experimental Section

### 2.1. Materials

PLA resin (4032D, Cargill Dow, Nature Works Co., Ltd., Blair, NE, USA), bamboo charcoal powder (200–325 mesh, carbonized at 650 °C for 2 h, Zhejiang Jizhu Biotechnology Co., Ltd., Zhenjiang, China), phosphoric acid (85 wt.%, AR), urea (99 wt.%, AR) and ethanol (99 wt.%, AR) were all applied as received without any purification.

### 2.2. Sample Preparation

#### 2.2.1. Synthesis of P-/N-Grafted Bamboo-Activated Carbon

Bamboo-activated carbon (BAC) was prepared from water steam activation of bamboo charcoal in a furnace at 900 °C for 2 h, and the steam pressure was set at 0.11 MPa. The as-prepared BAC samples were ultrasonically rinsed with D.I. water to remove possible dusts and ashes. After drying, BAC was blended with phosphoric acid (PA) at a 1:1 ratio in a three-neck flask. The blend was stirred at 70 °C for 30 min, then gradually heated to 100 °C and maintained for another 30 min. Continued the synthesis by adding urea solution to the blend, and let the PA/urea ratio be 4:1. The blend was slowly heated to 120 °C for 30 min and finally heated at 160 °C prior to produce foaming. Placed the foam in an oven at 105 °C for 6 h to obtain grafted bamboo-activated carbon (BACm), and foam was pulverized and ground into powders (200–325 mesh). The BACm was ready to use after rinsing several times with ethanol and drying at 100 °C for 2.5 h.

#### 2.2.2. Preparation of PLA Composite

PLA, BAC and BACm powders were dried prior to blending to prepared masterbatches at 180 °C via a regular twin screw extruder with the screw rotation speed at 30 rpm. The masterbatches were used to prepare specified composite samples by heat pressing at 180 °C. The formulations of BAC/PLA (as controls) and BACm/PLA composites were listed in Table 1. Neat PLA resin was also conducted as a reference.

### 2.3. Measurements and Characterizations

#### 2.3.1. BAC Characterization

Fourier transform infrared spectra (FTIR) of samples was determined with a spectrophotometer (Nicolet 6700, Thermo Fisher Scientific Inc., Waltham, MA, USA). The wave count range was set to 4000 to 400 cm^−1^.

The surface morphology of BAC and BACm were observed using field emission scanning electron microscopy (FE-SEM, SU8010, Hitachi, Tokyo, Japan) equipped with an energy dispersion analysis X-ray system (Octane plus, Ametek Inc., San Jose, CA, USA). The samples were sputtered with gold prior to measurements.

An X-ray diffraction (XRD) pattern was recorded by diffractometer (XRD-6000, Shimadzu, Kyoto, Japan) using Cu *Kα* radiation (λ = 1.542 Å) under a scanning step of 3°/min in a range of 10°–80°. All samples were prepared and analyzed at a 30 kV tube voltage and a 10 mA tube current.

The elemental analysis of after-combustion residue was determined by X-ray photoelectron spectroscopy (XPS, Thermo Fisher Scientific Inc., Waltham, MA, USA) with a non-monochromatic Al X-ray source (K-alpha 1486.68 keV) at a vacuum pressure below 10^−9^ mbar. The charge neutraliser was adjusted at 100 eV.

#### 2.3.2. Mechanical Properties

The mechanical properties of composite materials were tested using a microcomputer-controlled electronic universal testing machine (CMT610, MTS Industrial Systems, Shanghai, China) in accordance with China standards GB/T 1040 (tensile strength) and GB/T 9341 (flexural strength). For tensile testing, the width and thickness of the narrow stress zones in the samples were 5 mm and 2 mm, respectively. The gauge length was set to 60 mm and the loading speed was 2 mm/min. The sample size for flexural measurement was 80 mm × 10 mm × 4 mm, the loading span was set at 60 mm and loading speed at 2 mm/min.

#### 2.3.3. Thermal Performance

Thermal gravimetric analysis (TGA) was carried out using a thermogravimetric analyzer (F1, Netzsch Group, Selbu, Germany) under a nitrogen atmosphere. About 5–10 mg samples were analyzed in an aluminum crucible and tested at a heating rate of 10 °C/min under a nitrogen gas flow rate of 20 mL/min in the range of 35 °C to 800 °C. An empty crucible was used as a reference.

#### 2.3.4. Combustion Performance

Limiting Oxygen Index (LOI) was measured using the oxygen index meter (JF-3, Jiangsu Jiangning Analysis Instruments Company, Jiangsu, China) as the minimum concentration of oxygen required to initiate combustion in accordance with ISO 4589-2. The sample size was 100 mm × 10 mm × 4 mm.

Vertical flame testing was conducted using a Type 5402 vertical combustion tester in accordance with ASTM D3801. The sample size was 130 mm × 13 mm × 3 mm.

The combustion test was conducted in accordance with ISO 5660-1 using a cone calorimeter (CONE, FTT007, Fire Testing Technology, East Grinstead, UK), the sample size was 100 mm × 100 mm × 4 mm, irradiated at a heat flux of 35 kW/m^2^.

#### 2.3.5. Residue Carbon Analysis

Residue carbon recovered from combustion test was determined with a scanning electron microscope (SEM, TM3030, Hitachi, Tokyo, Japan) at an accelerating voltage of 15 kV. The specimen was sputtered by gold at a current of 15 mA.

## 3. Results and Discussion

### 3.1. Characterization of BAC and BACm

**Figure 1:**
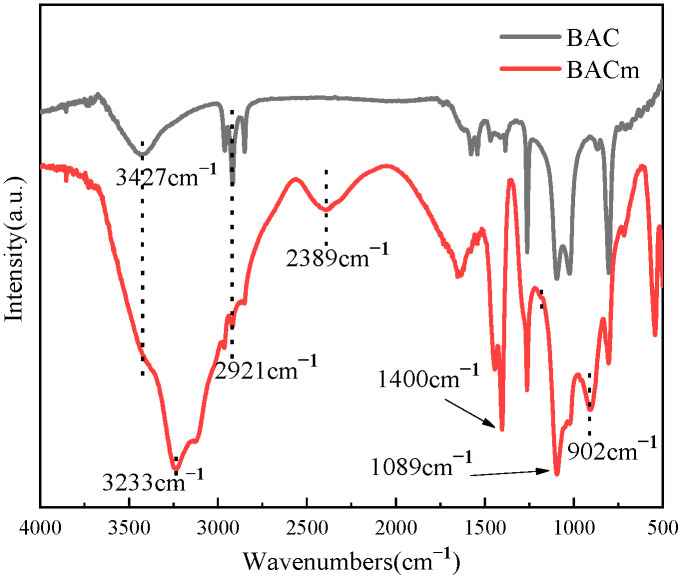
FTIR spectra of BAC and BACm.

The FTIR results of BAC and BACm were illustrated in Figure 1, and typical peaks of BAC were seen at 3427 cm^−1^ (–OH), 2921 cm^−1^ (C–H) and 1025 cm^−1^ (C–O) [38]. In contrast, after temperature controlled grafting with phosphoric acid and urea, characteristic peaks of hydroxyl and carboxyl groups intensified significantly and new peaks appeared for the BACm samples. The emerged peaks at 3131–3233 cm^−1^, 2389 cm^−1^, 1400 cm^−1^, 1089 cm^−1^ and 902 cm^−1^ were attributed to asymmetric stretching of –NH_4_, O–C–O, C–N bonds, symmetric stretching of P–O and P–O, respectively [39,40]. The appearance of these characteristic peaks proved the successful synthesis of P/N-containing samples.

**Figure 2:**
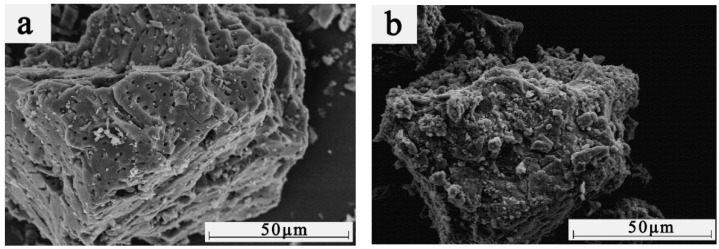

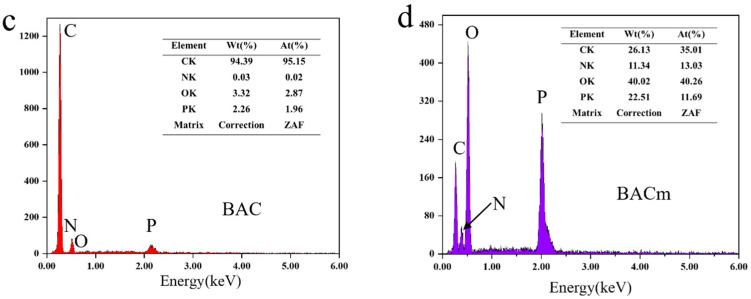
Surface morphology of BAC (**a**) and BACm (**b**) and EDAX spectra of BAC (**c**) and BACm (**d**).

The surface morphology and EDAX spectra of BAC and BACm were shown in Figure 2. It can be seen from Figure 2a that BAC had a highly developed pore structure and smooth surface, and its high specific surface area provided sufficient reactive sites for the grafting of flame retardant. Note that the amorphous surface of BACm existed with no micro-pores and was wrapped by flame retardant (Figure 2b). EDAX element analysis (Figure 2c or Figure 2d) showed the nitrogen, phosphorus and oxygen levels of the BACm increased substantially, which can be deemed as the grafting reaction between PA and urea with oxygen-containing functional groups of the BAC that had already been elucidated by FTIR data.

**Figure 3:**
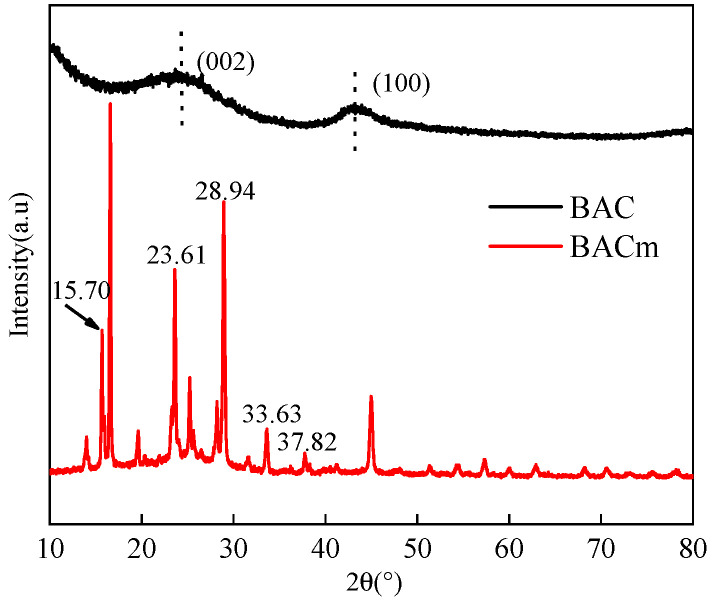
XRD patterns of BAC and BACm.

XRD analysis was employed to further verify the differences between BAC and BACm. As can be seen from the Figure 3, BAC did not show any sharp diffraction peaks, indicating the amorphous structure of carbon. The peaks of BAC at 2θ = 20°–30° represent carbon reflection from the stacked structure of the aromatic layer (002) [39,40,41,42,43], and the peak at 2θ = 43° indicated that pyrolytic carbon comprised of hexagonal structure (100) [44,45,46]. BACm had a series of crystal structures, e.g., 2θ = 15.7°, 23.61°, 28.94°, 33.63° and 37.82°, which suggested it had gained a crystal structure similar to the traditional flame retardant, ammonium polyphosphate(APP) [39,47,48,49], reassuring the successful grafting of P/N elements.

**Figure 4:**
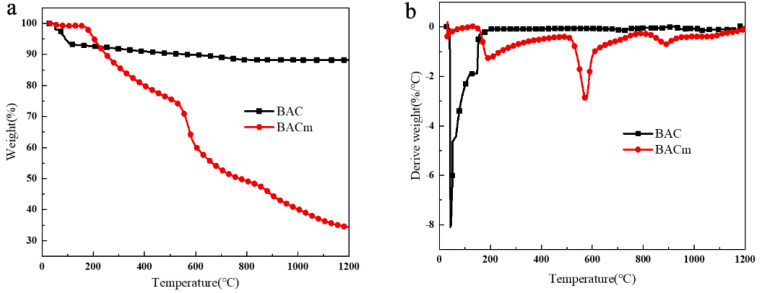
TGA (**a**) and DTG (**b**) curves for samples measured with a N_2_ atmosphere.

The TGA and DTG curves of BAC and BACm were depicted in Figure 4, and it was always easy to distinguish the pyrolysis differences between BAC and BACm. BAC experienced relatively small mass loss in the pyrolysis process, and the ultimate carbon residue rate ended up being 88.28 wt.%. The mass merely varied at a temperature of 200 °C and beyond, whereas on the other side, the carbon residue rate of BACm dramatically fell to 49.25 wt.%, and two main decomposition stages appeared, which were 150 °C–510 °C and 520 °C–1200 °C, respectively, corresponding to the evaporation and dehydration of water molecules and small molecules of phosphorus compounds, release of nitrogen-containing gases and further carbonation and thermal degradation of carbon residue in the process of heating up [50,51]. Equivalently, it was determined that gas-phase and condensate-phase flame retardants were generated during the combustion, which could also explain the reduction of BACm carbon residue compared to that of BAC.

### 3.2. Mechanical Properties

**Figure 5:**
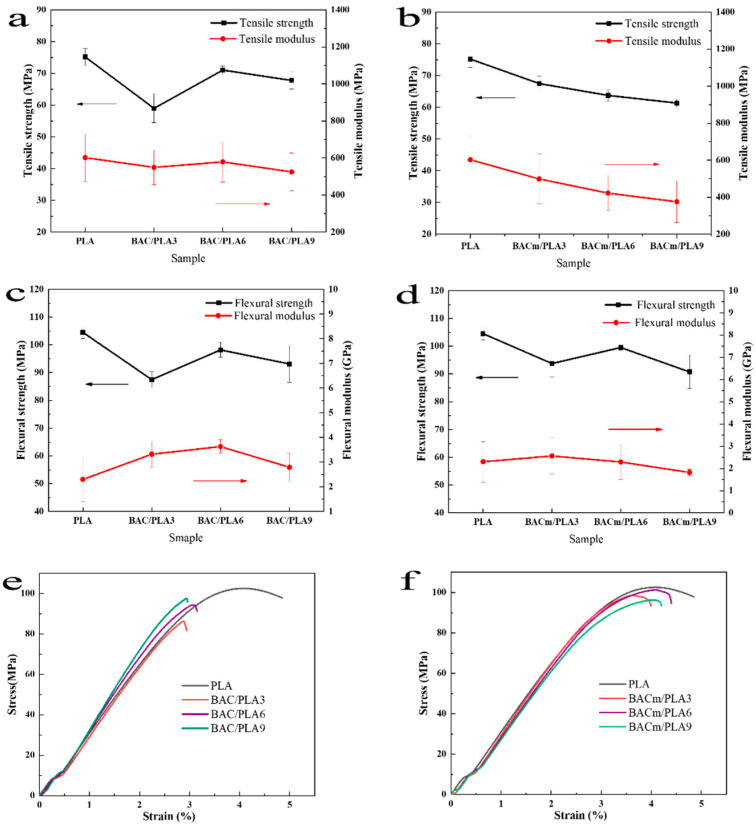
Mechanical properties of PLA composites (error bars represent standard deviation): BAC/PLA (**a**,**b**) and BACm/PLA (**c**,**d**), and typical tensile stress-strain curves of selected samples: BAC/PLA (**e**) and BACm/PLA (**f**).

The tensile and flexural strength of neat PLA and composites were shown in Figure 5, and the average and standard deviations were displayed in Table 2. In general, the addition of BAC or BACm to the composites was prone to lower the tensile and flexural strength of the composite. Low loading of BAC could presumably account for the tendency because BAC particles in the PLA matrix were scattered far away from each other and no workable mechanical interlocking occurred, which spontaneously hindered the stress propagation in the composites, and as a result, the tensile strength decreased. Whereas adequate addition of BAC can contribute to strength enhancement, however, when it reaches 9 wt.% in this work, a decrease in tensile strength can be seen, which may originate from stress concentration caused by the particle’s agglomeration in the composites. Similar results in flexural properties were exhibited for BAC/PLA composites, except that the flexural strength maximized when 6 wt.% BAC loading was applied.

The tensile strength variations of PLA/BAC-3/6/9 composites simply decreased with BACm loading, and the tensile strength decreased by 10%, 15% and 18%, in comparison with BAC/PLA counterparts in this work. From the data, it easy to know that the tensile strength dropped from 75.19 MPa to 61.30 MPa, and the tensile modulus from 602.49 MPa to 375.56 MPa, which signified rigidity reduction and deformation resistance enhancement owing to the BACm introduction into the composites.

Typical tensile stress-strain relationships for PLA/BAC and PLA/BACm composites were shown in the Figure 5e,f. Neat PLA posed with stronger non-linearity and ductility compared with the studied composites. BACm/PLA composites managed to overtake the BAC/PLA counterparts in ductility, which was approximate to that of neat PLA, the reason for which may lie in the fact that there is better compatibility of BACm with PLA, and the rough surface of BACm, or more functional groups conducive to the formation of hydrogen bonding and physical linkage with PLA molecular chain, eventually reduce the negative impact on the mechanical performance of the PLA matrix.

### 3.3. Combustion Performance

#### 3.3.1. LOI and UL94 Measurements

**Figure 6:**
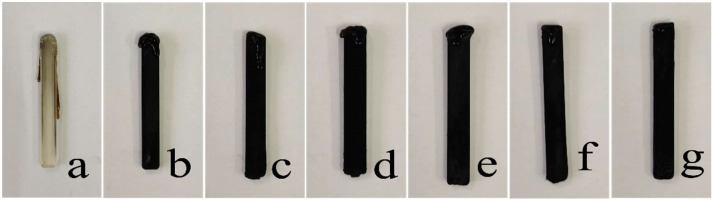
Digital images of composites after LOI tests: (**a**) PLA, (**b**) BAC/PLA3, (**c**) BAC/PLA6, (**d**) BAC/PLA9, (**e**) BACm/PLA3, (**f**) BACm/PLA6, (**g**) BACm/PLA9.

The limiting oxygen index (LOI) and UL-94 results were presented in Table 3. The images of PLA, BAC/PLA and BACm/PLA composites after LOI test were shown in Figure 6. It can be seen that neat PLA was easy to ignite, which found apparent dripping, and its LOI was 20.1% and no rated result was available in the UL-94 test. With regard to the BAC/PLA composites, it gained a notable enhancement in flame resistance, and the LOI increased with the BAC loading increment, whereas the dripping occurred to a limited degree. By contrast, the P-/N-modified BAC(BACm) revealed a remarkable improvement in flame retardancy when the LOI reached 28%; even for the composite with a small amount of addition of BACm at 3.0%, the value reached to 31.7%, and when the BACm was loaded at 9 wt.% in the current work, the dripping had been tremendously constrained, and a V-0 level had also been obtained in the UL-94 test. It can be concluded that the BACm exhibited a favorable property that outperformed the unprocessed BAC, even when applied at a quite low loading as a flame retardant in PLA composites.

#### 3.3.2. Cone Measurement

**Figure 7:**
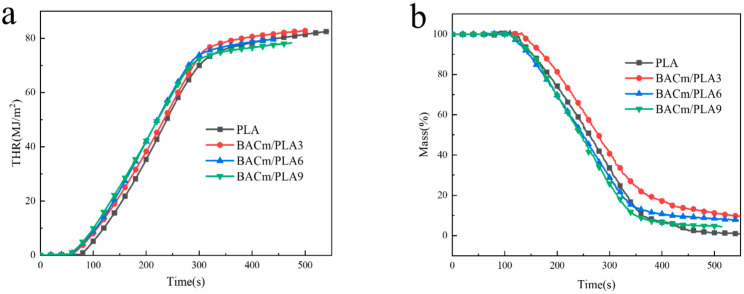

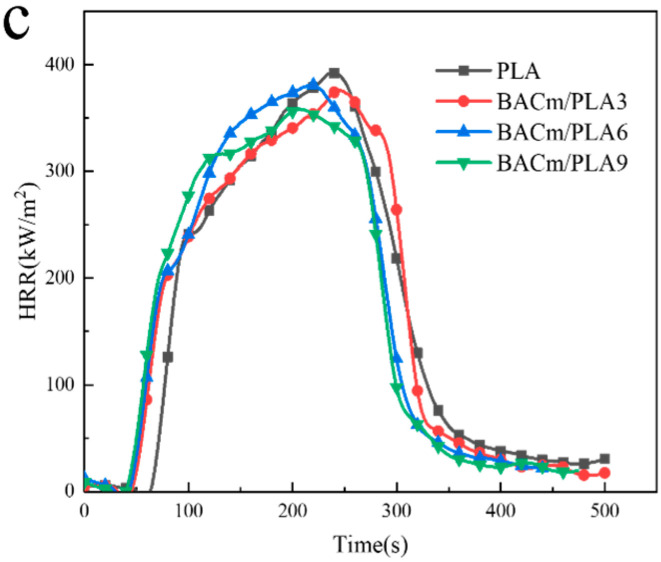
Cone analysis result of PLA composites: (**a**) THR, (**b**) mass loss of PLA, BACm/PLA3, BACm/PLA6 and BACm/PLA6, (**c**) HRR.

The heat release rate (HRR) and total heat release (THR) data of PLA composites were illustrated in Figure 7, and cone parameters were listed in Table 4. The BACm addition contributed an obvious time to ignite (TTI) move-up. TTI for neat PLA was approximately 67 s; nevertheless, the TTI immensely reduced to 42 s with a 9 wt.% loading of BACm into the PLA matrix, which possibly resulted from the phosphate release induced from the earlier decomposition of BACm. Simultaneously, it facilitated the PLA degradation that accentuated the flaming proceeding [52,53,54]. The high heat absorption rate of BACm contributed to rapid combustion and carbon layer emerging [53]. From Figure 7, it can be seen that THR and peak HRR (pHRR) for neat PLA were 82.63 MJ/m^2^ and 402.29 kW/m^2^, respectively, whereas they gradually descend with BACm loading growth, and they finally decreased to 78.26 MJ/m^2^ and 366.16 kW/m^2^, respectively. For BACm/PLA9, the heat release of all ignited samples fell in a range from 100 s to 300 s. Note that combustion residue was on an incessant rise, and the residual mass (RM) result attained an expected value of 8% for BACm/PLA9, but the peak mass loss rate (pMLR) decreased to 0.45 g/s, as compared to 0.59 g/s for that of neat PLA.

The CONE results also indicated the composites’ HRR and mass loss rate (MLR) had been superbly restrained, which proved to have a protective effect on the PLA matrix from flame interference. The emerging of a carbon layer built a robust firewall against the invasion of flame, oxygen and other flammable volatile gases. Nonetheless, inadequate loading of BACm in the composites in this work could not produce a continuous carbon layer, protecting PLA matrix as well.

### 3.4. Thermal Stability

**Figure 8:**
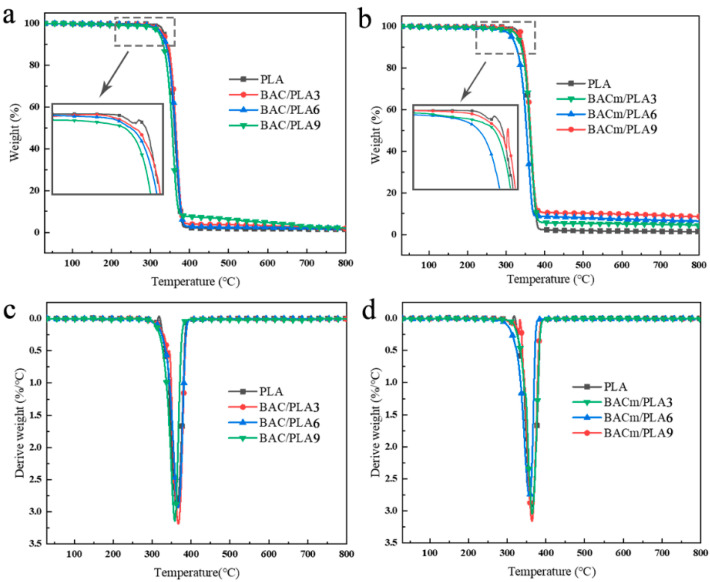
TGA (**a**,**c**) and DTG (**b**,**d**) curves for PLA composites.

Thermogravimetric analysis (TGA) results were listed in Table 5 and Figure 8. All samples presented a single thermal degradation phase but temperatures of 5% mass loss (T_5%_) or maximum temperatures of thermal degradation rate (T_max_) were marginally differed.

Neat PLA set about to quickly degrade at 322 °C, as recorded in Figure 8a, its T_max_ reached 363 °C and virtually all mass was lost at 400 °C. The corresponding carbon residue rate was determined at merely 1.35% at a terminal temperature of 800 °C; by contrast, acidic additive (phosphates) stimulated earlier decomposition of PLA composites when BACm was applied, which was in accordance with the previous TGA results. As seen in Table 5, the carbon residue rate increased progressively with BACm loading in the composites; notably, the actual rates of BACm/PLA composites attained a result of 4.44 wt.%, 6.42 wt.% and 8.60 wt.%, and outweighed 59.1%, 52.1% and 51.9% over that of the theoretical carbon residue rate results (2.79 wt.%, 4.22 wt.% and 5.66 wt.%, respectively, as calculated), which confirmed strengthened thermostability of composites with BACm application. The T_max_ barely differed for the neat PLA and BACm/PLA composites, which was, 363 °C, 364 °C, 357 °C and 363 °C, respectively. Thus it can be followed that BACm loading did not exert influence on the PLA degradation [51]. In the range of 600 °C to 800 °C, as seen in Table 5, the residue of PLA combustion saw no mass variation, yet a slight mass loss can be observed to a certain extent, and it demonstrated a postponed combustion of composites since the BACm addition.

### 3.5. Residual Carbon Analysis

**Figure 9:**
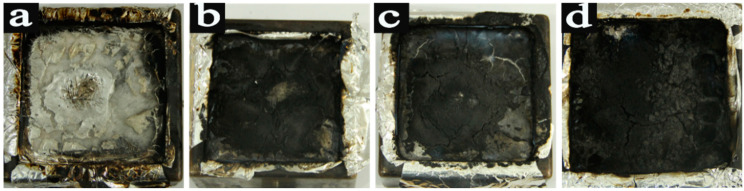
Digital photos of Cone-tested composites: PLA (**a**), BACm/PLA3 (**b**), BACm/PLA6 (**c**) and BACm/PLA9 (**d**).

The digital images of CONE-tested neat PLA and its composites were shown in Figure 9, and it was obvious that neat PLA practically took full combustion and residual carbon could be ignored. With the BACm loading to the composites, the residual carbon could be visually observed, and a non-continuous carbon layer can be found covering the sample surface when the BACm loading was less than 9 wt.% (Figure 9b,c). As a result, the layer could not be capable of forming an effective protective firewall against possible flame invasion. When sufficient BACm was loaded, the expansive residual carbon can be confirmed by its mass, continuity and compactness, which were superbly bettered. Generally, the expansive residual carbon can reduce the potential thermal and mass transfer between the condensed phase and the gas phase, which in return tended to induce combustion suppression [39].

**Figure 10:**
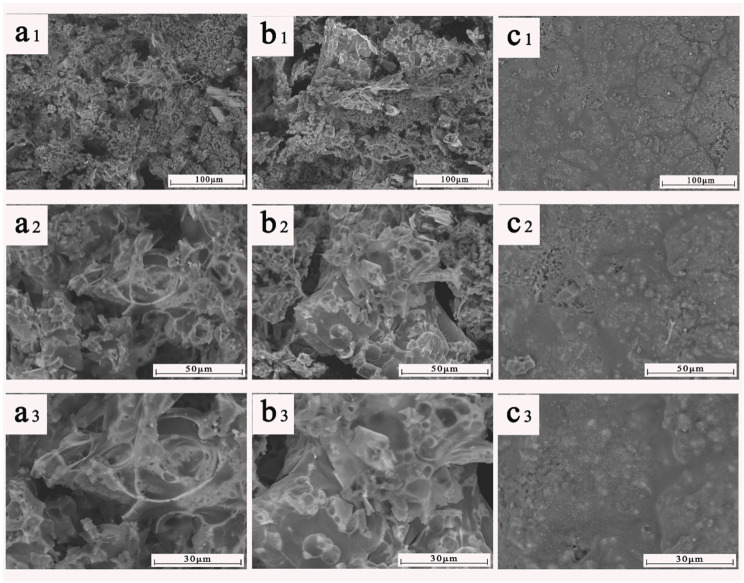
Morphology of residual carbon from CONE-tested composites: BACm/PLA3 (**a**), BACm/PLA6 (**b**) and BACm/PLA9 (**c**). 1, 2 and 3 were shown with different magnification.

Significant morphological difference of residual carbon can be noticed, as seen by the pattern in Figure 10. Voids and chasms were visible on the surface of BACm/PLA6 and BACm/PLA9 (Figure 10(a1,b1)), which could be exploited as the diffusion pathway of the heat and were volatile during combustion. In addition, by comparison, the BACm/PLA9 provided a fine wrinkled covering on the surface (Figure 10(c1)), which generated an efficient condensed phase flame retardancy, and moreover, wrinkle morphology can act as an enhancement framework for the composites in burning.

The residual carbon morphology of the sample after the cone calorimeter test was further studied by scanning electron microscopy and was shown in Figure 10. It can be seen that the carbon layer form was totally different. The surface of BACm/PLA3 or BACm/PLA6 (Figure 10(a1,b1)) was full of large holes and cracks that cannot be formed into intact continuous carbon layers, where heat and volatile pass easily through these layers, and are disabled to provide a good barrier to the underlying material during combustion. In contrast, when the addition of BACm reached 9 wt.% (Figure 10(c1)), the larger holes and cracks in the surface almost disappeared and a continuous and uniform carbon layer formed to guarantee a high-quality condensed phase flame retardant effect. As shown in Figure 10(a3), only 3 wt.% of BACm loaded to the composite, and a larger range of holes can be seen from the residue and the carbon layer was discontinuous. When 9 wt.% of BACm was loaded, the layer became quite dense, with only a small number of micropores, as presented in Figure 10(c3), and there were folds on the surface which can be used as support to strengthen the surface layer. A dense, continuous layer of carbon can act as an effective barrier deterring the transmission of combustible gases and heat between the combustion zone and the underlying matrix, thereby preventing further combustion of the matrix and obtaining better flame-retardant properties.

**Figure 11:**
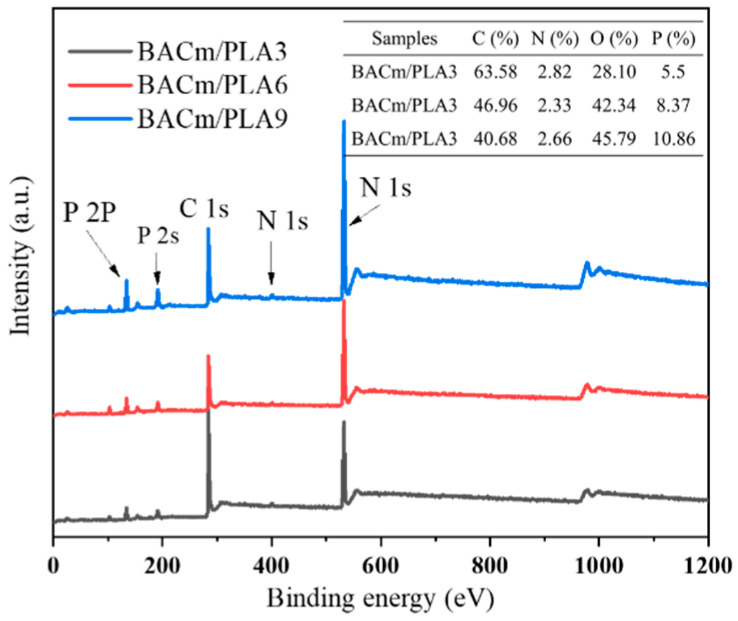
XPS spectra of the residue char of BACm/PLA composites after the cone calorimeter test.

The chemical components of the char residue after CONE testing were examined by XPS, and the corresponding spectrum were shown in Figure 11. The elements C and O constitute the majority of the residue, the total amount of both exceed 80%, followed by P and N elements. The contents of P, N and O elements increased with greater BACm addition in the composite as seen in the insert table of Figure 11.

**Figure 12:**
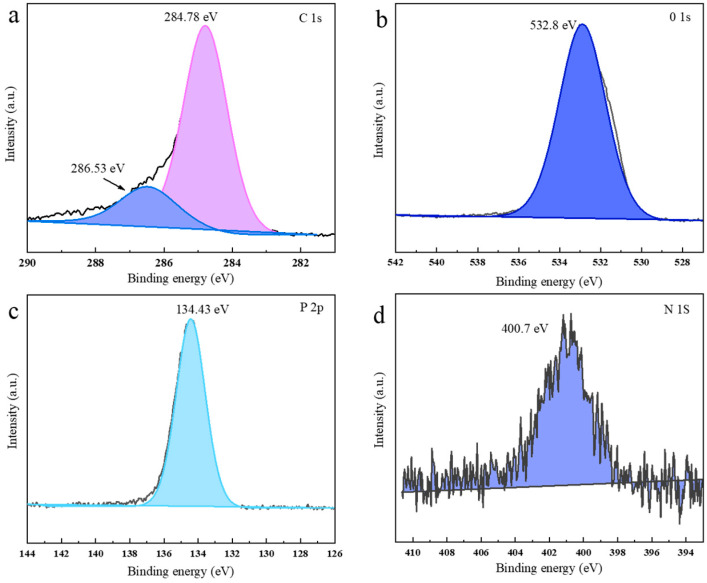
Spectra of the residue char of BACm/PLA9 for (**a**) C1s, (**b**) O1s, (**c**) P2p and (**d**) N1s.

Corresponding spectra were shown in Figure 12 for elements distributing on the surface of the carbon residue of BACm/PLA9. It was observed that the C1s spectrum in Figure 12a can be deconvoluted into two peaks at 284.7 eV and 286.5 eV, respectively, corresponding to C–C and/or C=C bonds in aliphatic or aromatic species and C–O in P–O–C groups, respectively. As shown in Figure 12b, the binding energy at 532.8 eV was assigned to –O– in C–O–C and/or C–O–P groups [55]. The peak at 134.4 eV of the P2p spectra in Figure 12c and 400.7 eV of N1s spectra in Figure 12d correspond to the P–O–P and the C–N bonds [56], respectively. The results show that BACm leads to the formation of a dense carbon layer containing P and N elements that are ultimately reflected in the detection of C–O–P, P–O–P and C–N bonds from the residue; namely, versatile flame retardancy of BACm in this work could be guaranteed based on the synergistical effect of P and N elements in the formulations.

## 4. Conclusions

In this work, a P-/N-containing bamboo-activated carbon (BACm) was formulated and applied in the flame retardancy enhancement of polylactic acid (PLA) composites. The analyses of FTIR, XRD, EDAX, XPS and SEM on the samples revealed a significant increase in nitrogen and phosphorus levels and the roughening surface morphology of BACm, which all confirmed the successful grafting of nitrogen and phosphorus to the carbon. TGA analysis implied that the thermal stability of BACm could be reinforced by grafting, and the carbon residue rate increased accordingly with BACm loading in the composites that could induce the early degradation and carbonation of PLA, and thus form more residual carbon. A mechanical strength study suggested a rigidity reduction and deformation resistance enhancement for BACm/PLA composites. The composites outperformed in flame retardancy as compared with neat PLA and BAC/PLA counterparts. When 9 wt.% BACm was loaded, the composites could attain a V-0 rating (UL94) with an LOI value at 31.75%. The CONE results demonstrated that the combustion performance would be improved by reaching lower THR, HRR and higher RM, which confirmed the BACm an effective flame retardant in PLA composites, and the residue carbon ratio outweighed the theoretical rate by more than 50%. The morphological structure of the carbon residue proved that BACm could promote forming compact and continuous carbon layers and effectively protect the underlying matrix away from combustion; further elemental analysis also showed residue with high levels of P and N elements and that BACm had a synergistical effect during combustion, which was most critical for the flame retardant property.

## Figures and Tables

**Table 1:** Formulations for PLA/BAC and PLA/BACm Composites.

Sample	Ingredients (wt.%)		
	PLA	BAC	BACm
Neat PLA	100.0	0	0
BAC/PLA3	97.0	3.0	0
BAC/PLA6	94.0	6.0	0
BAC/PLA9	91.0	9.0	0
BACm/PLA3	97.0	0	3.0
BACm/PLA6	94.0	0	6.0
BACm/PLA9	91.0	0	9.0

**Table 2:** Mechanical properties of PLA, BAC/PLA and BACm/PLA composites.

Sample	Tensile Strength (MPa)	Tensile Modulus (MPa)	Flexural Strength (MPa)	Flexural Modulus (GPa)
Neat PLA	75.19 ± 2.64	602.49 ± 128.7	104.51 ± 2.22	2.3 ± 0.91
BAC/PLA3	58.98 ± 4.52	549.25 ± 94.28	87.5 ± 2.86	3.32 ± 0.52
BAC/PLA6	71.04 ± 1.32	579.17 ± 108.38	98.18 ± 2.69	3.63 ± 0.27
BAC/PLA9	67.78 ± 2.74	525.04 ± 101.47	93.12 ± 6.56	2.79 ± 0.57
BACm/PLA3	67.48 ± 2.25	498.93 ± 135.07	93.77 ± 4.80	2.56 ± 0.81
BACm/PLA6	63.75 ± 1.75	422.37 ± 94.14	99.53 ± 0.93	2.29 ± 0.78
BACm/PLA9	61.3 ± 1.2	375.56 ± 112.1	90.76 ± 6.04	1.82 ± 0.14

**Table 3:** LOI and UL-94 results.

Samples	LOI (%)	UL-94	
		Dripping	Rating
Neat PLA	20.1	Yes	NR
BAC/PLA3	20.9	Yes	NR
BAC/PLA6	21.6	Yes	NR
BAC/PLA9	23.1	Yes	V-2
BACm/PLA3	28.0	Yes	V-2
BACm/PLA6	30.8	Yes	V-2
BACm/PLA9	31.7	No	V-0

**Table 4:** Cone parameters of PLA composites.

Sample	TTI (s)	THR (MJ/m^2^)	pHRR (kW/m^2^)	pMLR (g/s)	RM (%)
Neat PLA	67	82.63	402.29	0.59	0.92%
BACm/PLA3	47	82.78	386.29	0.63	4.56%
BACm/PLA6	45	79.76	390.45	0.68	7.74%
BACm/PLA9	42	78.26	366.16	0.45	7.99%

**Table 5:** Thermogravimetric analysis data of PLA composites.

Sample	T_5%_ (°C)	T_max_ (°C)	Carbon Residue Rate (wt.%)		
			400 (°C)	600 (°C)	800 (°C)
Neat PLA	334	363	2.28	1.63	1.35
BAC	90	45	90.97	89.85	88.28
BACm	203	574	79.98	60.49	49.25
BAC/PLA3	332	367	2.88	2.36	1.78
BAC/PLA6	313	368	4.01	3.16	1.81
BAC/PLA9	328	358	7.62	4.66	2.13
BACm/PLA3	329	364	5.77	5.15	4.44
BACm/PLA6	324	357	8.71	7.41	6.42
BACm/PLA9	323	363	10.59	9.78	8.60

## Data Availability

Not applicable.
